# Workflows and locations matter – insights from electronic hand hygiene monitoring into the use of hand rub dispensers across diverse hospital wards

**DOI:** 10.1016/j.infpip.2024.100364

**Published:** 2024-03-27

**Authors:** Christoph Senges, Christiane Herzer, Erlandas Norkus, Marco Krewing, Clara Mattner, Leonard Rose, Tobias Gebhardt, Frauke Mattner, Heide Niesalla

**Affiliations:** a HARTMANN SCIENCE CENTER, BODE Chemie GmbH a company of the HARTMANN GROUP, Hamburg, Germany; b GWA Hygiene GmbH, Stralsund, Germany; c Chair for Hygiene and Environmental Medicine, University Witten-Herdecke, Cologne Clinics, Cologne, Germany; d Institute of Rural Studies, Johann Heinrich von Thünen Institute, Braunschweig, Germany

**Keywords:** Hand rub dispensers/hand hygiene dispenser/hand disinfection dispenser/dispenser for hand disinfection/hand sanitizer, Hand hygiene compliance, Electronic monitoring system, Dispenser position/location, Healthcare-associated infections

## Abstract

**Background:**

While healthcare-associated infections (HAIs) affect approximately 3.2–6.5% of hospitalised patients in the US and Europe, improving hand hygiene (HH) could reduce HAI rates. Investigating HH is time-consuming and not always objective, and comprehensive, unbiased data is needed to develop effective strategies. Using electronic tools can provide new and detailed insights on the determinants of HH.

**Aim:**

To evaluate location-dependent usage of wall-mounted dispensers (WMDs) and point-of-care dispensers (POCs) using an electronic HH recording system.

**Methods:**

In this retrospective study, hand rub volumes were anonymously recorded for 931,446 disinfections from 17 wards in nine German hospitals using the electronic monitoring system NosoEx®. Number of disinfections and rub volumes of WMDs/POCs by ward and room type were analysed.

**Findings:**

Generally, WMDs were most prevalent. With >3 dispensers per bed and >20 disinfections per patient day, availability and use were highest in intensive care (ICU) and intermediate care (IMC), but here rub volumes from WMDs were lowest (∼2.0 mL). Although most dispensers are located in patient rooms (∼42%), they are more frequently used in hallways. In surgical ICUs, dispensers are often used in patient rooms, where contact with open wounds is common. About 3.6 mL of hand rub is used per disinfection in treatment rooms, the highest volume of all room types.

**Conclusion:**

Dispenser use was dependent on location, room type, ward specialisation and workflow. Optimising the location of hand rub dispensers (HRDs)s is not the only solution to improve HH, but can help reduce inconvenience, achieve more ergonomic workflows and better meet user needs.

## Introduction

Despite constant medical advances, the global burden of infectious diseases and associated deaths remains high [1]. Recently, the COVID-19 pandemic has brought this topic to an unprecedented level of public attention [2]. In addition to infections spreading among the general population, healthcare-associated infections (HAIs) are another major concern. In Germany, HAIs affected 4.6% of hospitalised patients in 2016 and 6.3% in 2020, while the overall prevalence in Europe is estimated at 6.5% (2016/2017) and in the United States at 3.2% (2015) [[3], [4], [5], [6]]. Consequences of HAIs include prolonged hospitalisation, increased morbidity and increasing consumption of antibiotics, which promotes the development of antibiotic resistance, leading to higher costs [[7], [8], [9]].

Since adequate hand hygiene (HH)—i.e. disinfecting hands correctly at the correct indications—is recognised as the most important measure to prevent HAIs, improving HH compliance (HHC; also called HH adherence) can reduce HAI rates significantly [[10], [11], [12], [13], [14]]. However, implementing HHC improvement strategies is a complex issue [15], which is further complicated by varying recommendations on rubbing techniques and ABHR volumes [[16], [17], [18], [19]]. Volumes required for correct disinfection—i.e. to cover the whole hand—are also affected by hand sizes, which puts the single-push strategy favoured by many healthcare workers into question [16]. Generally, the applied ABHR volume should not fall below 3 mL to ensure sufficient antimicrobial efficacy according to EN 1500 [20], while the World Health Organization (WHO) generally recommends disinfecting hands with ‘a palmful of ABHR’ [17].

Overall, HHC has been reported to range from 9.1% to 85.2%, depending on factors like geographic region and ward type [21]. Trying to improve HHC can be difficult, as it is influenced by both person-specific aspects (e.g., education, social norms, perceived infection risk, HH habits) and structural aspects (e.g., hospital type, numbers and locations of hand rub dispensers (HRDs)) [22,23]. Several interventions such as performance feedback or education have been shown to increase HHC rates, particularly when multimodal approaches were used, but HHC improvements are mostly not sustained due to effects like habituation [24,25]. An additional challenge in the investigation of HHC is the method of data collection: direct observation, the proposed gold standard [17], is susceptible to bias and inaccuracy. During direct observation, the personnel knowing to be observed is usually more compliant (Hawthorne effect), observations cannot be performed everywhere 24/7, observer-dependent recording biases may occur, etc. [[26], [27], [28], [29], [30]].

A more recent development is the use of electronic tools [31,32], which allow a different perspective on fundamental determinants of HHC, as they enable hospital-wide 24/7 monitoring without an omnipresent human observer. Data gathered with such systems can be used to optimise workflows and reduce inconveniences like suboptimal HRD localisation, a major factor limiting HHC [[33], [34], [35]]. Generally, a sufficient number of well-accessible HRDs is desirable [23]. While no international recommendations regarding the minimal number of HRDs exist [23], the Commission for Hospital Hygiene and Infection Prevention (KRINKO) at the Robert Koch Institute (RKI) in Germany recommends one HRD per bed in intensive care units (ICUs) and 0.5 per bed in general wards close to patient beds [36]. However, practice has shown that two HRDs per bed, placed in visible and easily accessible positions, might be optimal. [23,33,35,[37], [38], [39]]. Beyond that, it is necessary to consider the workflow of diverse healthcare activities when placing HRDs, as adequate placement of HRDs is more important than their number [37,39]. This is also in line with economic considerations: ‘as many as necessary, as few as possible’. There are no official guidelines for optimal location but in practice most hospitals provide fixed mounted HRDs near doors, sinks, or beds as well as pocket HRDs [23].

In this retrospective study, we evaluated the location-dependent usage of wall-mounted dispensers (WMDs) and point-of-care dispensers (POCs) by means of an automated HH recording system, operated in nine hospitals. Since the use of automated monitoring systems can initially arouse scepticism among the staff [32], the study was conducted over a longer period and was accompanied by onboarding of the personnel. On multiple wards covering various disciplines in several hospitals, we investigated HRD localisation between and within room types as well as frequency and quality of usage.

## Methods

### Study design

This was a retrospective observational study conducted in nine hospitals in Germany from January 1, 2022, to July 30, 2022 (211 days). These healthcare facilities include four primary care hospitals, three standard care hospitals, and two maximum care hospitals. The 17 observed wards included six ICUs (two of them surgical intensive care units), three intermediate care (IMC), three orthopaedic and general surgery (including one neurosurgery ward), two neurology, and three ‘other’ wards (oncology, dementia, and ear/nose/throat (ENT)).

### Participants

As every use of each HRD was recorded, healthcare workers (HCWs), other staff, patients, and visitors all contributed to the data collected here. Data were collected in an anonymous way without revealing the identity of the participants. To introduce the electronic HH monitoring system to the HCWs, an initial presentation was given by a local hygiene specialist with support of the manufacturer. Additionally, printed materials covering frequently asked questions were distributed. The local hygiene specialists visited wards regularly to explain the system and answer questions during the study.

### Electronic hand hygiene monitoring system

The electronic HH monitoring system NosoEx® (GWA Hygiene GmbH, Stralsund, Germany; [31,40]) consisted of sensors and data hubs. Pre-existing HRDs (either wall-mounted or point-of-care) were equipped with sensors that detected: i) the number of actuations; and ii) the delivered volume of ABHR per disinfection. Multiple consecutive HRD actuations were considered as one hand disinfection, incorporating the combined volume of each actuation. While sensors on WMDs (i.e., fixed, difficult-to-relocate HRDs but, for technical reasons, also HRDs permanently installed on nursing carts) used magnetic detection, sensors on POCs (i.e., mobile HRDs in holders) used pressure detection. The localisation of mobile POCs was not tracked after the initial installation. When the sensors detected activity, data of HRD usage were recorded anonymously by the sensor module and were forwarded via the NosoEx® network to the data hub. The data hub collected all data and transferred them to a database on a server of a certified data centre. Access to the data and evaluation on a monitor was only possible via encrypted connections. HRD locations were recorded on digital maps of ward layouts.

### Data protection, recording and analysis

Prior to installation, the consent of the workers council and, if necessary, the facility's data protection officer was obtained. Data on HRD usage were recorded anonymously in a ward-wide manner. Maps of wards shown in the following are recreations of actual ward layouts to ensure the anonymity of involved hospitals and wards.

The NosoEx® system was installed in three of the 17 wards as part of a larger study with GWA Hygiene GmbH and BODE Chemie GmbH, a company of the HARTMANN GROUP. For all other wards, the hospitals themselves decided to install the digital monitoring system and which dispensers to fit with sensors. In most cases, existing dispensers in established locations were fitted with sensors. The ABHR used was recorded during installation. However, it was not influenced or tracked subsequently. A wide range of different professional ABHRs were used, the three most common being Desderman® pure (Schülke & Mayr GmbH, Norderstedt, Germany), Sterillium® classic pure (BODE Chemie GmbH, a company of the HARTMANN GROUP, Hamburg, Germany) and Promanum® Pure (B. Braun Melsungen AG, Melsungen, Germany). Data on the use of sensor-equipped HRDs were automatically and anonymously recorded each time a dispenser was used. As no comprehensive user information was recoded across all 17 wards, it is not possible to distinguish between users or categories of users, and staff, patients and visitors all contribute to the recorded data. However, we assume that most users are staff members. Actuations recorded by the monitoring system are translated into dispensed volumes and summarised into disinfections using proprietary algorithms. For analysis, this information was aggregated into a single dataset, exported from the database and anonymised. This raw data was made available to the two industry partners and the academic partners. Analyses were either performed jointly or cross-checked by all partners involved. As comprehensive location information was not available for all dispensers and patient days were not always available for all wards on all days, the figure texts below indicate which data were used in the analyses. Outliers that were obviously incorrect, e.g. a single disinfection with 143.55 mL ABHR, were excluded from the analysis.

### Statistical analysis

Descriptive data analysis was performed by comparing the number of disinfections or ABHR volumes of either WMDs or POCs by ward type and medical department. Microsoft Excel (ver. 2304, Microsoft, Redmond, Washington, USA) and Python (ver. 3.9) with pandas (ver. 1.2.5), numpy (ver. 1.23.4), matplotlib (ver. 3.6.0) and seaborn (ver. 0.12.1) were used for analysis. The Mann-Whitney U test was performed manually in Excel.

## Results

In total, we analysed data from 931,446 disinfections from a total of 1,082 sensor-equipped HRDs. For 1,002 HRDs, their location was also available. Patient days were available for 828,716 disinfections, and both patient days and HRD location were available for 704,971 disinfections.

### HRD availability and ABHR volumes by ward and HRD type

**Figure 1:**
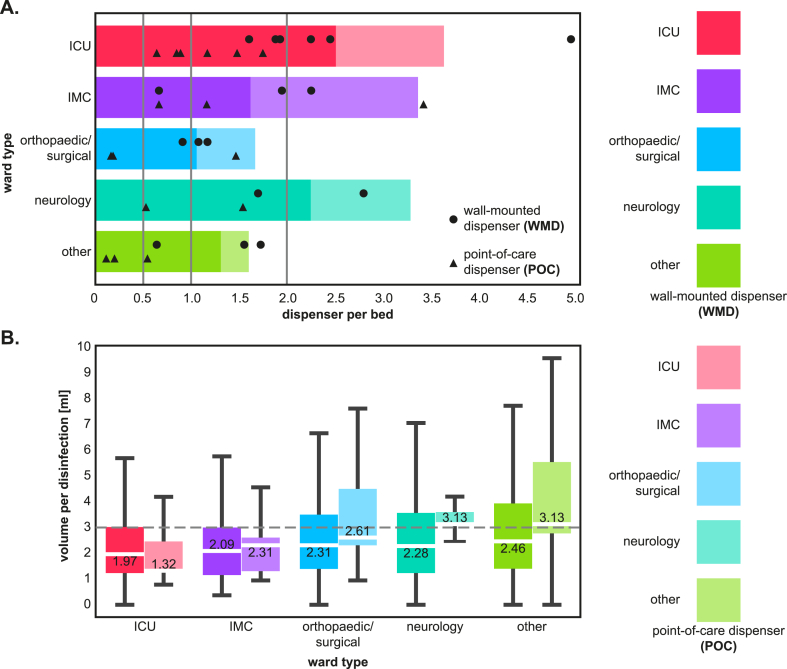
(**A**) Mean number of HRDs per bed by ward type and comparison of POC and WMD numbers on individual wards. Bars show the averages of POCs and WMDs per ward type, while circles (WMD) and triangles (POC) show the individual numbers of HRDs per ward. Vertical lines indicate recommendations of German KRINKO (0.5 HRDs/bed on normal wards and one HRD/bed on ICU) and the number considered optimal (two HRDs/bed) [23,39]. (**B**) Volume of ABHR used per disinfection for WMD vs. POC by ward type. The whiskers of the boxplot represent minima and maxima, while the boxes cover the first and third quartiles. Outliers are excluded. White lines and numbers in boxes mark the median. Gray dashed horizontal line indicates recommended minimal volume (3 mL) [20]. Data from the entire observation period. Strong colours represent WMD and pale colours POC. ABHR, alcohol-based hand rub; HRD, hand rub dispenser; ICU, intensive care unit; IMC, intermediate care; POC, point-of-care dispenser; WMD, wall-mounted dispenser.

HRD availability differed markedly between ward types, with the highest availability on ICUs (>3.5 per bed), closely followed by IMC (∼3.4 per bed) and neurology wards (∼3.3 per bed), for which mean numbers all exceeded the numbers considered optimal (i.e., around two per bed [23,39]) (Figure 1A). Although orthopaedic/surgical as well as ‘other’ wards had the lowest number of HRDs (∼1.6 per bed) and remained below the optimum of 2 HRDs per bed, all wards were equipped with more HRDs than officially recommended by the KRINKO to be placed near patient beds (i.e., one per bed in ICUs, 0.5 per bed in general wards [36]).

When considering the individual wards, one of the six ICUs had 5 WMDs per bed, more than all other wards (Figure 1A). The highest number of POCs (∼3.4 per bed) was provided by one of the three IMC wards. While ratios often vary, overall most of the 17 participating wards were equipped with more WMDs than POCs (Figure 1A). POC numbers averaged about 60.6 ± 50.8% (mean ± SD) of WMD numbers, and only three wards had more POCs than WMDs.

Across all ward types, ABHR median volumes were mostly below the recommendation of 3 mL per application, which is already considered insufficient for larger hands. Only POCs on neurology and ‘other’ wards had median volumes per application (3.13 mL) above the recommended 3 mL. Overall median volumes ranged from 1.32 mL (POCs on ICU) to 3.13 mL (POCs on neurology and ‘other’ wards). Marginally higher ABHR volumes were used from POCs, possibly related to different volumes dispensed or the fact that POCs are often used in direct context with a care activity (Figure 1B).

### Disinfections per patient day

**Figure 2:**
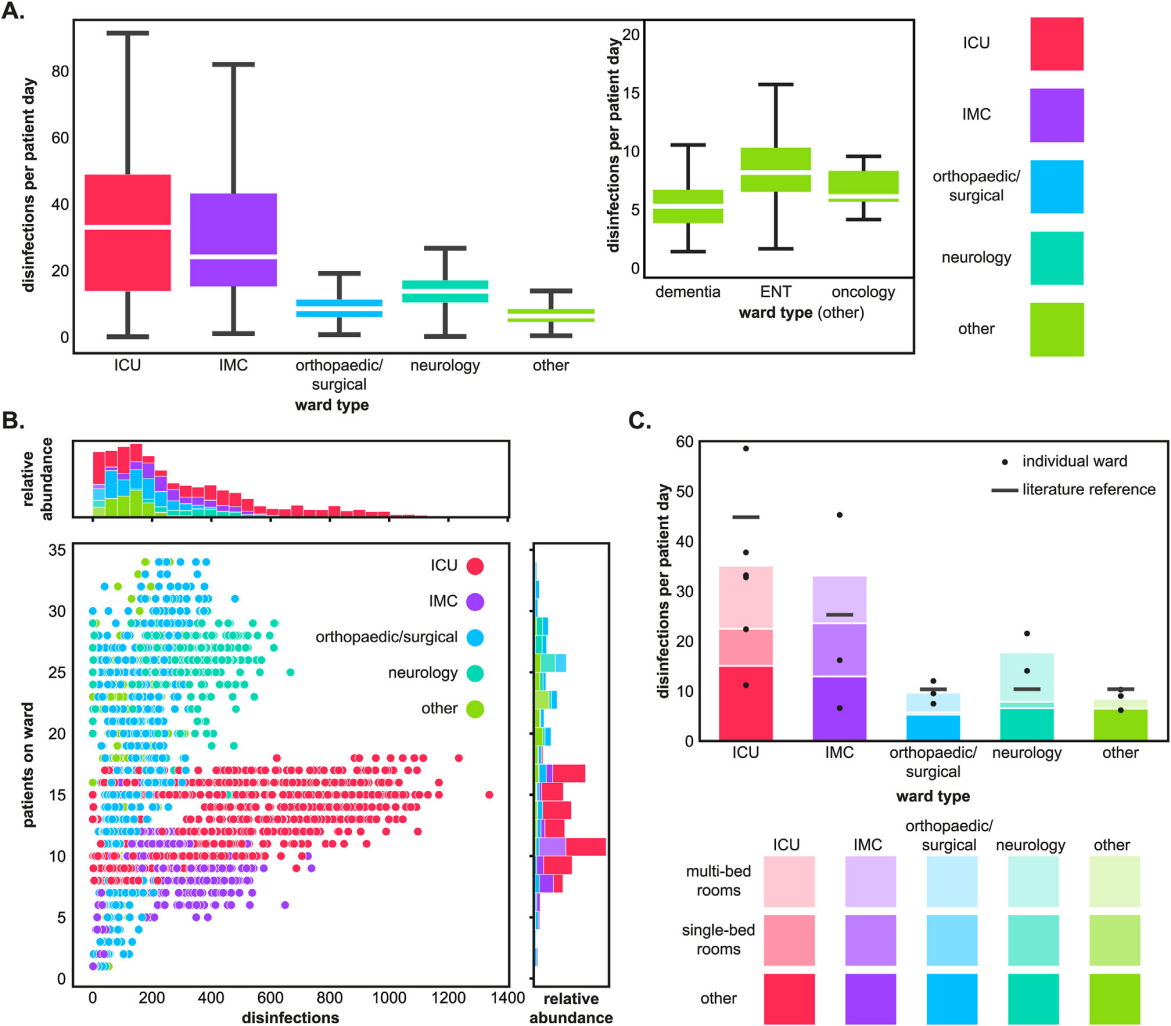
(**A**) Number of disinfections per patient day by ward type. Median values: ICU 32.85; IMC 24.10; orthopaedic/surgical 8.51; neurology 13.76; other 6.29. The whiskers of boxplots represent minima and maxima, while the boxes cover the first and third quartiles. Outliers are excluded. White lines mark the median. (**B**) Disinfections per number of patients on ward. Each dot represents the number of inpatients and disinfections for one day of data collection. Example: Ward 1 had 200 disinfections and 15 patients on day 1 = dot at x-axis 200, y-axis 15; 400 disinfections and 17 patients on day 2 = further dot at x-axis 400, y-axis 17; etc. (**C**) Ward-related comparison of the arithmetic mean of disinfections per patient day in multi-bed rooms vs. single-bed rooms vs. other rooms. Black dots represent averages of individual wards, black lines indicate ward specialisation-specific averages reported by the German National Reference Center for the Surveillance of Nosocomial Infections (NRZ) for 2022 [41]. Sum of averages: ICU 35.14; IMC 32.67; orthopaedic/surgical 9.67; neurology 17.66; other 8.41. Reference data: ICU 45; IMC 25; orthopaedic/surgical 10; neurology 10; other 10 [41]. A and B are based on disinfections with patient day information, whereas C is based on disinfections with patient day and location information. Pale colours represent multi-bed rooms, intermediate colours single-bed rooms and strong colours other rooms on the ward. ENT, ear/nose/throat; ICU, intensive care unit; IMC, intermediate care.

On ICUs and IMC wards, hand disinfections were on average performed more frequently than on orthopaedic/surgical, neurology, and ‘other’ wards (Figure 2A–C), with the difference between ICU/IMC wards vs. neurology/orthopaedic/surgical/‘other’ wards being statistically significant according to the Mann-Whitney *U*-test (z-value: -29.87; *P*-value: 4.8 x 10^−105^). With a median number of 32.85 disinfections per patient day, ICUs ranked highest, followed by IMC wards with 24.10 disinfections per patient day. Numbers on neurology, orthopaedic/surgical, and ‘other’ wards ranged from 6.29 to 13.76 disinfections per patient day. However, the variability of the data was also much greater for ICUs and IMC wards than for all other ward types (Figure 2A). Among the ward types summarised as ‘other’, the ENT ward had the highest number of disinfections per patient day. The dementia ward, where usually only few invasive care activities are carried out, had the lowest number of disinfections per patient day overall (Figure 2A).

The number of patient days was more variable on orthopaedic/surgical, neurological, and ‘other’ wards than on ICUs and IMC wards (Figure 2B). While it varied from 1-34 patient days for orthopaedic/surgical and ‘other’ wards, ICUs and IMC wards showed steadier occupancies and accommodated fewer patients, which is typical for these ward types. Interestingly, the numbers of daily disinfections were still higher (up to 1,000 on days with 12–18 patients) on ICUs and IMC wards than on orthopaedic/surgical, neurological, and ‘other’ wards with more patients, where disinfections per day increased with patient load. Here, the highest numbers of disinfections (>400) were only seen on days with ≥20 patients (Figure 2B).

Comparing the arithmetic means of HRD usage per patient day, most disinfections were performed on ICUs, followed by IMCs (Figure 2C). Most hand disinfections per patient day are performed in multi-bed rooms and outside of patient rooms (referred to as ‘other’ in Figure 2C). For instance: in ICUs single-bed rooms, the average was 7.42 hand disinfections per patient day, whereas in multi-bed rooms and outside of patient rooms, the averages were 12.78 and 14.94, respectively. On orthopaedic/surgical and ‘other’ wards less than one hand disinfection on WMDs per patient day was observed in single-bed rooms. Interestingly, only IMC and neurology wards surpassed their respective ward-specific average reported by the German National Reference Center for the Surveillance of Nosocomial Infections (NRZ) for 2022 [41]. On IMC wards, 32.67 hand disinfections per patient day were observed, compared with a national average of 25, while on neurology wards, an average of 17.66 hand disinfections per patient day were observed, compared with a reported average of 10 [41]. It should be noted that the data on disinfections per patient day were ward-specific and not room-specific and, thus, may have been affected by differences in occupancy (e.g., preferred use of multi-bed rooms, or single-bed rooms being used as isolation rooms).

### HRD use is location-dependent

**Figure 3:**
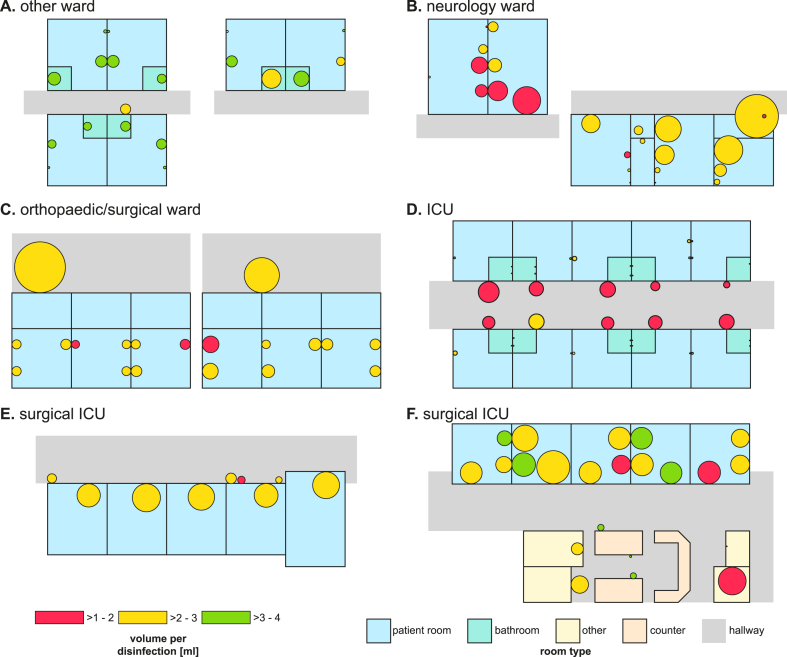
HRD usage on ward maps. All maps are exemplary, anonymised recreations of ward layouts. (A, B) Dispenser usage when multiple dispensers are available in patient rooms. (C, D) Dispenser usage on hallways and in patient rooms on orthopaedic/surgical wards and non-surgical ICUs and (E, F) on surgical ICUs. All subfigures based on disinfections with location information. Circles represent WMDs and always touch the wall they are mounted to. Larger circles indicate more frequent usage, but circle sizes are not comparable between subfigures. Green/yellow/red circles indicate that on average >3–4 mL/>2–3 mL/>1–2 mL of ABHR were used per disinfection, respectively. ABHR, alcohol-based hand rub; HRD, hand rub dispenser; ICU, intensive care unit; WMD, wall-mounted dispenser.

Depending on the ward specialisation, HRD usage preferences varied (Figure 3). When multiple HRDs were available in patient rooms of non-surgical and non-ICU wards, the ones closest to the door were used most frequently (Figure 3A, B). In wards that are not surgical ICUs, HRDs in the hallway were used most frequently (Figure 3C, D) as most transit usually occurs in hallways. On operative ICUs, where many procedures require on-site hand disinfection (e.g., wound care), HRDs in patient rooms were used most frequently (Figure 3E, F).

HRDs were mostly but not exclusively available and in use in hallways and patient rooms (Table I). Overall, the greatest proportion of HRDs was located in patient rooms (41.8%), hallways (16.2%), and bathrooms (15.9%) whereas the fewest HRDs were found in operating rooms (0.1%) and locker rooms (0.4%). Regarding the total number of disinfections, permanently installed HRDs (i.e., WMDs) were most often used in break rooms (12,036 ± 7,135) and technical rooms (10,881 ± 8,698), while portable HRDs (i.e., POCs) were most frequently used in work rooms (5,444 ± 3,810) and hallways (3,985 ± 2,026), which may be due to routine disinfection before breaks, transit, etc. Notably, the greatest ABHR volume per disinfection (3.59 mL) was consumed in treatment rooms, which are areas with little probability of use by patients but with direct patient contact, thus requiring hand disinfection according to the WHO recommendations on HH moments [17]. In contrast, the lowest ABHR volumes were used in technical rooms (2.25 mL) and break rooms (2.36 mL) where non-patient and treatment-independent activities are usually carried out.

**Table I:** Distribution and usage of HRDs by room type across all included wards. For disinfections with location information, means and standard deviations are given by HRD type (WMD vs. POC). For disinfection output, mean volumes are given. POC dispensers were mobile and may have been repositioned to other room types during data collection. Of the WMDs dispensers assigned to technical and treatment rooms, 16 and 7, respectively, were permanently installed HRDs on nursing carts. Disinfections per WMD/POC refers to the total number of disinfections recorded during the observation period. ABHR, alcohol-based hand rub; POC, point-of-care dispenser; SD, standard deviation; WMD, wall-mounted dispenser

Room types	Dispenser distribution, %	Number of rooms, n	Number of WMDs, n	Number of POCs, n	Disinfections per WMD, mean ± SD	Disinfections per POC, mean ± SD	ABHR volume per disinfection, mL
Patient room	41.8	189	326	93	7,939 ± 3,241	1,629 ± 1,065	2.71
Hallway	16.2	40	129	33	8,373 ± 4,135	3,985 ± 2,501	2.59
Bathroom	15.9	133	159	0	3,797 ± 3,205	-	3.00
Work room	6.4	37	45	19	5,887 ± 3,110	5,444 ± 3,810	2.72
Treatment room	3.8	20	34	4	2,239 ± 1,182	1,178 ± 707	3.59
Storage room	3.6	22	26	10	2,958 ± 1,107	1,073 ± 698	3.13
Lock to operating room	2.9	24	29	0	2,975 ± 499	-	2.60
Office	2.3	19	23	0	8,357 ± 8,250	-	2.44
Technical room	2.1	4	21	0	10,881 ± 8,698	-	2.25
Kitchen	2.0	11	19	1	5,474 ± 4,463	7 ± 4	2.64
Break room	1.3	9	12	1	12,036 ± 7,135	1,980 ± 885	2.36
Cleanroom	1.3	9	13	0	2,658 ± 1,446	-	2.53
Locker room	0.4	4	4	0	868 ± 301	-	2.60
Operating room	0.1	1	1	0	702 ± 0	-	2.53

## Discussion

Performing HH correctly at the indicated moments is considered the most important strategy to reduce HAIs [[42], [43], [44]]. Improving HHC lowers infection rates, and since HAIs are associated with increased morbidity, mortality, length of stay, and costs, investments in improving HHC can pay off quickly [8,[45], [46], [47], [48]]. Despite years of research with various intervention studies, HHC remains a global issue. Many factors influencing HHC—from personnel education to constructional aspects of a hospital—have been identified, a fundamental one being convenience [49]. Improved HRD usability, visibility, and accessibility are associated with higher compliance [34,50]. Our data support these findings. In this study, we investigated availability- and location-dependent HRD use using a digital monitoring system, which—unlike direct observation—might not prompt personnel to use HRDs in less favourable positions. While electronic monitoring does not allow an assessment of whether HH is being performed at the correct indications, the work of Diefenbacher *et al.* [51] showed that the number of dispenser uses and HHC are highly correlated.

As electronic monitoring becomes more widespread, it is being used more frequently in studies and interventions to monitor or improve HH [52,53]. While the installation of a digital monitoring system can have a positive effect as an intervention in itself [53], many studies use electronic monitoring for real-time feedback, often with very positive results [52,54,55]. Looking at the intervention literature as a whole, the majority of recent interventions have been multimodal [[56], [57], [58], [59]], mostly focusing on education and training, feedback, reminders, and provision of HH materials and infrastructure [56]. Multimodal interventions can be highly successful, as in the study by Medeiros *et al.*, in which HHC was improved from 27% to 58% [60]. However, multiple simultaneous interventions often make it difficult to assess the role of individual aspects such as infrastructure improvement (e.g. dispenser locations) in improving HH. The importance of dispenser locations was recently supported by a study in which optimised dispenser locations led to an improvement in ABHR consumption from 20.6 mL/patient day to 25.3 mL/patient day [61].

### Number of HRDs not the only decisive factor

The sheer number of available HRDs is not the only factor influencing HHC. While, for example, Boog *et al.* [49] described that an increase from 0.25 HRDs/bed to 1 HRD/bed resulted in an increase in compliance from 41% to 48% after patient contact, HHC improves only up to a certain threshold when more HRDs are installed [39,49]. All ward types investigated here fulfilled the German KRINKO recommendation on HRDs per patient bed (Figure 1A). On average, more HRDs were available on ICU, IMC, and neurology wards than on orthopaedic/surgical and ‘other’ wards. And while ICU and IMC wards had significantly higher than average use of HRDs, neurology wards had slightly more disinfections than orthopaedic/surgical or ‘other’ wards (Figure 2A).

Generally, ICU and IMC wards are known for increased ABHR use compared to general wards. For example, data on ABHR consumption of the German National Reference Center for the Surveillance of Nosocomial Infections (NRZ, HAND-KISS module) for 2022 reveal an average use of 128 mL/patient day on ICUs, 68 mL/patient day on IMCs, and only 29 mL/patient day on general wards (31 mL/patient day on neurology wards) [41]. Interestingly, orthopaedic/surgical wards, which are often prone to SSIs, have comparatively few HRDs and low HRD usage, an observation also described in the literature [62].

Except for IMC and neurology wards, all other wards displayed slightly lower rates of hand disinfections per patient day compared to the data reported by the German NRZ (Figure 2C). The NRZ data relies on disinfectant purchases and is calculated using a generalisation of 3 mL/hand disinfection, whereas the data obtained from the electronic system is based on the consumption of disinfectant at the moment of use. This eliminates the influence of replacing partially empty bottles of ABHR or sharing among wards, leading to more precise measurements. Additionally, factors such as empathy can explain differences in the utilization of HRDs between wards: as previously reported, an empathetic focus on particularly vulnerable patient groups is probably more pronounced among paediatricians or geriatricians than, for example, surgeons with a more technical focus and could contribute to better HHC to protect these patients [51]. Regarding location, we found that usage is mostly focused on those HRDs closest to the door if several HRDs are available within one room (Figure 3A, B), which is consistent with observations of others [49,63].

### HRD usage depends on room types and HRD location

HRDs are most often positioned in patient rooms, bathrooms, and hallways (Table I). This is in line with current recommendations and observations: many healthcare-related activities are performed in the patient room and HRDs should be positioned close to the point of care [38,64,65]. Historically, HRDs are often located near sinks, similar to soap dispensers for handwashing, and standardised positions can improve HRD use [66]. HRDs in hallways are passed by everyone moving through the hospital, making them regular reminders that can influence HH behaviour positively [67] (Figure 2C). Most POCs were initially located in patient rooms (Table I) and were therefore easily accessible to practice HH during care activities, as frequently recommended [64,65]. As expected, in multi-bed rooms, where the presence of several patients warrants more care activities, and in hallways more hand disinfections are performed (Figure 2C). While HRDs in hallways can be conveniently used in a walk-by, there are generally few indications for HDs in hallways, especially if patient room doors are closed, so these disinfections may contribute little to infection prevention.

### Dispensers are used where needed

Whether and how HRDs are used often depends on the workflows performed in a room. HRDs are frequently used in high-traffic areas like hallways (Figure 2, Figure 3C, D). Usage in patient rooms, however, seems to be workflow-dependent as the most frequent use here was documented on surgical ICUs, possibly because of frequent contact with open wounds (Figure 3E, F). Generally, HCWs tend to perform hand disinfections most reliably after contact with potentially infectious material [[68], [69], [70]]. Consistently, we observed in the group of ‘other’ ward types that the one with the greatest risk of exposure to infectious material (ENT) had the highest number of disinfections, while the one with the lowest exposure risk (dementia) showed the lowest number (Figure 2A). Accordingly, we observed the largest ABHR volumes per disinfection in treatment rooms, where many healthcare-related procedures take place (Table I). It is possible that one of the factors influencing ABHR volume is the time available for HH, especially since many HCWs report to not have enough time for proper hand disinfection [64]. Usually, there is enough time for proper hand disinfection before or after interacting with patients or using the restroom. HRDs in hallways might be used by visitors or without an indication when passing by. Generally, it is important to offer multiple HRDs in several configurations to promote HHC as preferences of HCWs differ and the use of a larger number of dispensers can help overcome structural obstacles. However, targeted optimisation of locations has been shown to improve ABHR consumption [61].

### Limitations

Although the digital monitoring system facilitates recording data without direct observers, it also adds limitations that may have impacted our data. For example, HCW behaviour might be affected by the sensors on HRDs as they are reminders of the monitoring. Digital monitoring also has disadvantages compared to direct observation: disinfections recorded here cannot be linked to HH indications, making it difficult to assess the impact on infection prevention. The data presented here is a snapshot in time; changes in ABHRs used, dispenser locations or ward layouts have not been continuously tracked. In addition, technical problems may have led to gaps in data collection, therefore not all dispenser uses may have been recorded. The current system did not allow to track the localisation of POC dispensers and HRDs on mobile carts, certain data could therefore not be evaluated room-specifically; however, technical solutions should enable automatic tracking of mobile HRDs in the future. In addition, we do not have detailed records of the location of the dispensers, for example whether they are in line of sight or concealed. As no comprehensive user information has been recoded across all wards, we cannot distinguish between categories of users, therefore staff, patients and visitors all contributed to the data. In addition, the use of HRDs by non-healthcare personnel and visitors, as well as maintenance activities, can affect the data unevenly. In the future, the use of transponders based on user categories will help to overcome this limitation. Regardless of the system, it should also be taken into account that the group summarised as ‘other wards’ was heterogeneous and that the occupancy of multi-/single-bed rooms could only be recorded at ward level. Moreover, the data were collected during the COVID-19 pandemic, which drastically influenced all healthcare activities as well as ward occupancy and staffing ratios [71].

## Conclusions

Our findings suggest that HRD use is affected by room types, ward types and workflows. Thus, optimising HRD location cannot serve as the sole solution to improve HHC. Although more research is required to draw firm conclusions, the following general recommendations can be derived from our data:

• Exact positioning of HRDs should be considered. It is not effective to simply overload patient rooms with HRDs.
• Near-patient HRDs should be provided if taking care of patient wounds or other aseptic procedures are a main activity. HRDs close to doors and in hallways that can be used on the fly may be more practical on wards with less invasive key activities.
• Using a digital monitoring system gives more precise consumption data than relying solely on ABHR purchases, especially when location information is included to separate disinfections near patients from those that contribute little to infection prevention.
• Other behaviour-influencing factors such as knowledge, training, and empathy also play important roles for HHC and should not be neglected.

To allow drawing more specific recommendations for different occupational groups and typical room constellations, future research should include tracking of POCs, tracking HRD usage of different HCW groups, and defining typical room configurations to comparatively examine the influence of HRD positions and types.

## Conflict of interest statement

CS, HN and MK are employees of BODE Chemie GmbH, a company of the HARTMANN GROUP. CH, TG and EN are employees of GWA Hygiene GmbH. The Cologne hygiene team (including CM, LR and FM) provides hygiene supervision for some of the wards included in this study. BODE Chemie GmbH, a company of the HARTMANN GROUP, provided financial support for the project in Cologne, including a staff position for a NosoEx® study as well as an additional study.

## Funding statement

This work was supported by BODE Chemie GmbH, a company of the HARTMANN GROUP, and GWA Hygiene GmbH.

## Ethics statement

Not required. We have not worked with personal or patient information, nor have we influenced activities on the wards.
